# Measuring the performance of HIV self‐testing at private pharmacies in Kenya: a cross‐sectional study

**DOI:** 10.1002/jia2.26177

**Published:** 2023-10-17

**Authors:** Katrina F. Ortblad, Benn Kwach, Shengruo Zhang, Magdalene Asewe, Patricia Atieno Ongwen, Rachel C. Malen, Kendall Harkey, Josephine Odoyo, Paul Gathii, Greshon Rota, Monisha Sharma, Daniel Knight Were, Kenneth Ngure, Victor Omollo, Elizabeth Anne Bukusi

**Affiliations:** ^1^ Public Health Sciences Division Fred Hutchinson Cancer Center Seattle Washington USA; ^2^ Centre for Microbiology Research Kenya Medical Research Institute Nairobi Kenya; ^3^ Department of Epidemiology University of Washington Seattle Washington USA; ^4^ Jhpiego Nairobi Kenya; ^5^ Department of Global Health University of Washington Seattle Washington USA; ^6^ School of Public Health Jomo Kenyatta University of Agriculture and Technology Nairobi Kenya; ^7^ Department of Obstetrics and Gynecology University of Washington Seattle Washington USA

**Keywords:** HIV self‐testing, differentiated care, HIV prevention, PrEP, sensitivity, specificity

## Abstract

**Introduction:**

HIV self‐testing (HIVST) has the potential to support daily oral pre‐exposure prophylaxis (PrEP) delivery in private pharmacies, but many national guidelines have not approved HIVST for PrEP dispensing. In Kenya, pharmacy providers are permitted to deliver HIVST, but often do not have the required certification to deliver rapid diagnostic testing (RDT). We estimated the performance of provider‐delivered HIVST compared to RDT, the standard of care for PrEP delivery, at private pharmacies in Kenya to inform decisions on the use of HIVST for PrEP scale‐up.

**Methods:**

At 20 pharmacies in Kisumu County, we trained pharmacy providers (pharmacists and pharmaceutical technologists) on blood‐based HIVST use and client assistance (if requested). We recruited pharmacy clients purchasing sexual and reproductive health‐related products (e.g. condoms) and enrolled those ≥18 years with self‐reported behaviours associated with HIV risk. Enrolled clients received HIVST with associated provider counselling, followed by RDT by a certified HIV testing services (HTS) counsellor. Pharmacy providers and clients independently interpreted HIVST results prior to RDT (results interpreted only by the HTS counsellor). We calculated the sensitivity and specificity of pharmacy provider‐delivered HIVST compared to HTS counsellor‐administered RDT.

**Results:**

Between March and June 2022, we screened 1691 clients and enrolled 1500; 64% (954/1500) were female and the median age was 26 years (IQR 22–31). We additionally enrolled 40 providers; 42% (17/40) were pharmacy owners and their median years of experience was 6 (IQR 4–10). The majority (79%, 1190/1500) of clients requested provider assistance with HIVST and providers spent a median of 20 minutes (IQR 15–43) with each HIVST client. The sensitivity of provider‐delivered HIVST at the pharmacy was high when interpreted by providers (98.5%, 95% CI 97.8%, 99.1%) and clients (98.8%, 95% CI 98.0%, 99.3%), as was the specificity of HIVST in this setting (provider‐interpretation: 96.9%, 95% CI 89.2%, 99.6%; client‐interpretation: 93.8%, 95% CI 84.8%, 98.3%).

**Conclusions:**

When compared to the national HIV testing algorithm, provider‐delivered blood‐based HIVST at private pharmacies in Kenya performed well. These findings suggest that blood‐based HIVST may be a useful tool to support PrEP initiation and continuation at private pharmacies and potentially other community‐based delivery settings.

## 1 INTRODUCTION

HIV self‐testing (HIVST) is a relatively new technology that has been shown to increase recent HIV testing in diverse populations and settings and facilitate linkage to prevention and treatment interventions [1, 2, 3]. HIVST may be particularly useful for expanding access to and continuation on daily oral pre‐exposure prophylaxis (PrEP); a highly effective HIV prevention intervention [4, 5] that requires testing every 3 months to detect incident HIV acquisitions [6, 7]. The necessity for frequent testing while using PrEP creates delivery challenges for health systems (e.g. costs and over‐burdened providers) [8, 9, 10] and access challenges for clients (e.g. stigma associated with HIV clinics and time spent waiting at clinics) [8, 9, 10], which contribute to limited utilization of PrEP services among those who could benefit most [11, 12]. HIVST may help overcome some of these challenges by enabling community‐based PrEP delivery models outside clinic settings (e.g. online delivery [13] and pharmacy delivery [14]) and increasing efficiencies within clinic‐based PrEP delivery models (e.g. testing while waiting [15] and multi‐month dispensing supported with interim HIVST [16]).

However, in most settings, HIVST is recommended as a screening tool only [6, 7]—not to be used to inform PrEP prescribing or dispensing, which limits its ability to support health systems and clients. Concerns with using HIVST to support PrEP delivery are primarily related to the slightly lower sensitivity and specificity of HIVST (especially oral‐fluid HIVST) compared to rapid diagnostic testing (RDT) and the potential for user error in HIVST administration and interpretation [17]. Lower HIVST field performance can lead to inappropriate PrEP delivery to persons with undiagnosed HIV, potentially resulting in the development of PrEP‐related drug resistance, which can compromise the effectiveness of some first‐line antiretroviral treatment (ART) regimens. Thus, RDT delivered by a certified provider is recommended as the standard‐of‐care for PrEP dispensing [6, 7].

Recommending the use of RDT for PrEP delivery, however, limits the expansion of PrEP delivery in community‐based settings, where few individuals are certified to complete RDT. For example, at private pharmacies, which are a promising new setting for PrEP service delivery in Africa [18, 19]. Recent pilot studies in Kenya found that pharmacy‐delivered PrEP services are in demand and may reach individuals with HIV risk indication who are not already engaged in clinic‐based PrEP services (e.g. older men) [20, 21]. Moreover, pharmacy‐delivered PrEP services can achieve comparable PrEP continuation levels to those observed in public clinics [20, 21, 22]. However, to conduct RDTs for PrEP delivery, Kenyan pharmacy providers have to complete a time‐consuming 2‐week RDT certification programme [23]. Additionally, RDT provision at the pharmacy may take time away from routine service delivery. HIVST may be more feasibly implemented than RDT in settings such as pharmacies by reducing personnel time associated with HIV testing and enabling greater client privacy, as users can conduct their own self‐test [24, 25, 26].

In this study, we sought to quantify the performance of pharmacy provider‐delivered blood‐based HIVST compared to RDT completed by certified providers at private pharmacies in Kenya. The findings from this study may be utilized to inform HIV testing guidelines for oral PrEP delivery at pharmacies and other community settings as well as potentially for new longer‐acting PrEP modalities (e.g. bi‐monthly injections [27, 28] and monthly vaginal rings [29, 30]).

## 2 METHODS

### 2.1 Study design and setting

We conducted a cross‐sectional study at 20 private pharmacies in Kisumu, Kenya. Kisumu, an urban city located in Kisumu County (western Kenya), has a population‐level HIV prevalence of 17.5%; one of the highest in Kenya [31]. In Kisumu County, private pharmacies are ubiquitous, with 27 hospitals, 7 wholesale and 119 licensed community pharmacies in 2022 [32].

To be eligible for study selection, pharmacies had to be licensed with the Kenya Pharmacy and Poisons Board, have a full‐time licensed provider (i.e. pharmacists or pharmaceutical technologists), have a private room for HIV counselling and testing, and be willing to participate in research activities. Additionally, pharmacies had to agree to have a research assistant, who also served as a Kenya National AIDS and STI Control Programme‐certified HIV testing services (HTS) counsellor, stationed full‐time at the pharmacy for questionnaire completion and HIV RDT provision.

### 2.2 Participants and recruitment

We enrolled pharmacy providers and clients. Pharmacy providers were eligible for participation if they were ≥18 years old and willing to participate in research activities. All providers completed a 1‐day training on blood‐based HIVST, which included training on how to counsel clients on HIV risk and prevention behaviours, advise clients on use and interpretation of HIVST kits, and guide clients on linkage to treatment and prevention services, including PrEP, as needed.

Pharmacy clients were eligible for participation if they were ≥18 years old, self‐reported at least one behaviour associated with HIV risk or a recent HIV exposure and were willing to participate in research activities, including two forms of HIV testing. To assess HIV risk, we used Kenya's eight‐item (modified to nine‐item) Rapid Assessment Screening Tool, which asks individuals about their behaviours in the past 6 months (e.g. engagement in transactional sex) and is routinely used at public clinics to inform PrEP eligibility [33]. To assess recent HIV exposure, we asked if clients had sex with someone who may be living with HIV, had experienced sexual assault or were exposed to blood or bodily fluids in the past 72 hours. Pharmacy providers recruited participants by asking clients seeking services and products associated with potential HIV risk (e.g. emergency contraception and treatment for sexually transmitted infections) if they would be interested in study participation and referring those interested to the research assistant.

This study protocol was approved by the institutional review board at the Fred Hutchinson Cancer Center and the scientific ethics review unit at the Kenya Medical Research Institute. All participants completed written informed consent (available in English, Dholuo and Kiswahili) and were compensated 500 Kenyan Shillings (∼$4.50 US Dollars) for their time completing research activities.

### 2.3 Procedures

Enrolled pharmacy clients were asked to complete two HIV tests in a private pharmacy room: (1) provider‐delivered blood‐based HIVST, and (2) HTS counsellor‐administered blood‐based RDT. HIV testing and pre‐ and post‐test counselling were provided to participants free of charge.

First, pharmacy providers explained HIVST use and results interpretation to clients who completed HIVST alone but were encouraged to ask providers for assistance if needed. We used the Mylan HIV Self‐Test (Mylan Pharmaceuticals Private Limited), which has a sensitivity of 99.8% (95% confidence interval [CI] 98.7%, 100.0%) and specificity of 99.8% (95% CI 99.1%, 100.0%) in controlled environments [34]. To facilitate the HIVST process, clients and providers had access to the Mylan self‐test written and pictorial instructions [35] as well as a simple flip book (i.e. job aid), similar to those used to facilitate RDT at public clinics. Following HIVST, we asked pharmacy clients and providers to interpret the self‐test result separately, and then discuss the result together.

Next, clients completed an RDT administered by the HTS counsellor, mirroring the test administration utilized at public clinics in Kenya for PrEP dispensing. We used the Determine HIV‐1/2 test (Abbott Diagnostic Medical Co. Ltd), which has a sensitivity of 99.9% (95% CI 99.4%, 100.0%) and specificity of 100.0% (95% CI 98.0%, 100.0%) in controlled environments [36]. If clients tested negative, they were referred to clinic‐based PrEP or post‐exposure prophylaxis (PEP) services. If clients tested positive or the RDT test result differed from the provider‐interpreted HIVST result, the HTS counsellor followed national testing guidelines [37] and completed a second RDT using the First Response HIV 1–2.0 Card test (Version 2.0, Premier Medical Corporation Private Ltd), which has a sensitivity of 100.0% (95% CI 99.2%, 100.0%) and specificity of 100.0% (95% CI 99.5%, 100.0%) [38]. If any RDT was positive, pharmacy clients were referred to an HIV clinic for confirmatory testing and ART. If the second RDT was negative, the research assistants collected a dried blood spot from clients for laboratory‐based HIV polymerase chain reaction (PCR) testing; all clients were informed by the research staff of their PCR test results, when available.

### 2.4 Data collection

Research assistants with experience in HIV prevention research completed quantitative questionnaires with pharmacy providers and clients. Providers (two per pharmacy) were asked about their demographics and the provision of products related to HIV testing. Clients were asked about their demographics, behaviours associated with HIV risk (self‐reported), preferences for HIVST prior to testing and perceptions of provider‐delivered HIVST at pharmacies. During the time clients completed HIVST, research assistants recorded observations including whether clients requested assistance from providers with the HIVST process (and for what steps). Following HIVST, research assistants recorded providers’ and clients’ interpretations of the HIVST result and documented the result with a photo. All data were captured electronically using CommCare (Dimagi).

### 2.5 Outcomes

We measured the performance of provider‐delivered blood‐based HIVST when interpreted by both pharmacy providers and clients. Our primary outcome was the sensitivity and specificity of provider‐interpreted HIVST compared to HTS counsellor‐interpreted RDT, the “gold standard” (i.e. the test being used to support PrEP dispensing at public clinics). We also measured the sensitivity and specificity of client‐interpreted HIVST compared to the gold standard as well as the negative and positive predictive values (i.e. NPV and PPV) of provider‐ and client‐interpreted HIVST compared to RDT.

For discrepant HIVST and RDT test results, we measured the percentage of poor HIVST performance attributable to misinterpretation of results. For discrepant tests, two authors (KFO and SZ) reviewed images of HIVST results uploaded to CommCare and interpreted them following the HIVST kit manufacturer's guidelines (e.g. one bold line indicates strong negative, two bold lines indicate strong positive, one bold and one faint line indicates weak positive). We used the researchers’ interpretations of the HIVST results as the reference to identify which discrepant HIVST and RDT results were attributable to provider or client misinterpretation versus differences in kit performance.

We also measured clients’ experiences with and perceptions of HIVST at the pharmacy. Specifically, we assessed whether clients requested assistance with HIVST (using research assistant observations), their linkage to care plans following HIV testing and their perceived acceptability of HIVST at the pharmacy. For the acceptability assessment, we asked questions that measured different component constructs of acceptability (e.g. self‐efficacy, burden and perceived effectiveness) outlined in the Theoretical Framework of Acceptability (TFA) [39, 40]. If >80% of clients “agreed” or “strongly agreed” with a statement assessed using a 5‐point Likert scale, we categorized that acceptability construct as acceptable [36]. Additionally, for clients who tested HIV negative and were interested in initiating PrEP services, we asked them where (e.g. public clinic and private pharmacy) they would most prefer accessing PrEP.

### 2.6 Analyses

We used descriptive statistics to measure most study outcomes among all participants and by sex groups. To estimate uncertainty around our HIVST sensitivity and specificity outcomes, we calculated 95% binomial CIs. With a sample of 1500 pharmacy clients and a population‐level HIV prevalence of 5%, we estimated we could measure 95% HIVST sensitivity with 0.05 precision. We used Stata/MP 14.0 (College Station) for all analyses.

## 3 RESULTS

**Figure 1:**
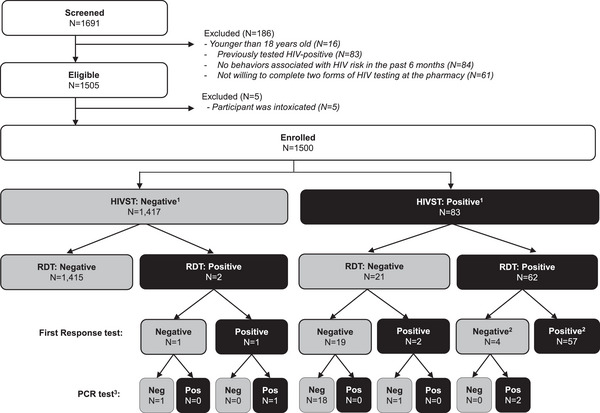
CONSORT diagram.

From March to June 2022, we screened 1691 individuals, identified 1505 eligible pharmacy clients and enrolled 1500 (see Figure 1). Among the 186 clients excluded, 45% (84/186) reported no behaviours associated with HIV risk, 45% (83/186) had previously tested HIV positive and 33% (61/186) were not willing to complete two forms of HIV testing. During this same period, 40 pharmacy providers were enrolled and completed questionnaires.

The median age of enrolled pharmacy clients was 26 years (interquartile range [IQR] 22–31 years) and years of school was 14 (IQR 11–16) (see Table 1). Most clients were female (64%, 954/1500) and reported one (primary) sexual partner (53%, 794/1500); nearly half reported casual sexual partners (42%, 627/1500). The most common behaviours associated with HIV risk were inconsistent condom use (87%, 1310/1500) and sexual partners of unknown HIV status (41%, 609/1500) (full list in Table 1). One in 10 clients reported condomless sex with someone who may be living with HIV in the past 72 hours (12%, 173/1500). Approximately one‐third of clients reported ever HIVST (32%, 456/1500); most clients reported a preference for blood‐based versus oral‐fluid HIVST (83%, 1251/1500) and for accessing HIVST at pharmacies versus clinics (89%, 1327/1500). Client characteristics and preferences were largely consistent across sex groups, except for multiple sexual partners, which was more commonly reported among male clients (*p*<0.001).

**Table 1:** Characteristics of the enrolled pharmacy clients and providers, *n*/*N* (%)

Pharmacy clients	All, *N* = 1500	Men, *N* = 546	Women, *N* = 954
**Demographics**			
Age, median (IQR)	26 (22, 31)	27 (23, 34)	25 (22, 30)
Sex: Female	954/1500 (64)	0/546 (0)	954/954 (100)
Years of school, median (IQR)	14 (11, 16)	14 (12, 16)	13 (11, 15)
Monthly income (In Kenyan Shillings)	10,000 (5000, 20,000)	10,000 (5375, 20,000)	10,000 (5000, 20,000)
Relationship status			
*One primary partner only*	794/1500 (53)	211/546 (39)	583/954 (61)
*One primary partner and casual partners*	351/1500 (23)	187/546 (34)	164/954 (17)
*Casual partner(s) only*	276/1500 (18)	120/546 (22)	156/954 (16)
*Single*	75/1500 (5)	26/546 (5)	49/954 (5)
**Behaviours associated with HIV risk**			
Kenya's HIV RAST; behaviours in the past 6 months^a^			
*Sexual partner(s) living with HIV with risk of transmission*	90/1500 (6)	45/546 (8)	45/954 (5)
*Inconsistent condom use*	1310/1500 (87)	465/546 (85)	845/954 (89)
*Sexual partner(s) with unknown HIV status*	609/1500 (41)	262/546 (48)	347/954 (36)
*Multiple sexual partners*	510/1500 (34)	260/546 (48)	250/954 (26)
*Exchanged sex for money/gift*	285/1500 (19)	127/546 (23)	158/954 (17)
*Diagnosed with or treated for a sexually transmitted infection (STI)*	149/1500 (10)	68/546 (13)	81/954 (9)
*Used PEP more than twice*	99/1500 (7)	52/546 (10)	47/954 (5)
*Forced to have sex/physically assaulted*	75/1500 (5)	26/546 (5)	49/954 (5)
*Shared needles during intravenous drug use*	26/1500 (2)	20/546 (4)	6/954 (1)
Potential recent HIV exposure, in the past 72 hours^b^			
*Unprotected sex with someone who may be living with HIV*	173/1500 (12)	79/546 (15)	94/954 (10)
*Experience sexual assault*	14/1500 (1)	2/546 (0)	12/954 (1)
*Exposed to someone's blood/body fluids*	324/1500 (22)	135/546 (25)	189/954 (20)
**PrEP and HIVST use history**			
Ever used PrEP	92/1500 (6)	18/546 (3)	74/954 (8)
Ever used HIV self‐test	456/1500 (32)	156/546 (30)	300/954 (32)
HIV self‐test preference: type			
*Blood‐based*	1251/1500 (83)	445/546 (82)	806/954 (85)
*Oral‐fluid*	240/1500 (16)	95/546 (17)	145/954 (15)
*Don't know*	6/1500 (0)	5/546 (1)	1/954 (0)
*Neither method*	3/1500 (0)	1/546 (0)	2/954 (0)
HIV self‐test preference: access location			
*Pharmacy*	1327/1500 (89)	483/546 (89)	844/954 (89)
*HIV clinic*	110/1500 (7)	36/546 (7)	74/954 (8)
*STI clinic*	3/1500 (0)	3/546 (1)	0/954 (0)
*Other outpatient clinic*	57/1500 (4)	21/546 (4)	36/954 (4)
HIV self‐test preference: utilization location			
*Pharmacy*	690/1500 (46)	255/546 (47)	435/954 (46)
*HIV clinic*	60/1500 (4)	20/546 (4)	40/954 (4)
*STI clinic*	0/1500 (0)	0/546 (0)	0/954 (0)
*Other outpatient clinic*	19/1500 (1)	8/546 (2)	11/954 (1)
*Home*	729/1500 (49)	261/546 (48)	468/954 (49)

**Pharmacy providers, *N* = 40**	**All,** ***N* = 40**	**Men,** ***N* = 24**	**Women,** ***N* = 16**
**Demographics**			
Age, median (IQR)	31 (27, 37)	32 (27, 39)	31 (26, 37)
Sex—Female	16/40 (40)	0/24 (0)	16/16 (100)
Own the pharmacy	17/40 (42)	12/24 (50)	5/16 (31)
Years in profession, median (IQR)	6 (4, 10)	5 (4, 10)	6 (3, 9)
**Service provision**			
Days worked at the pharmacy per week, median (IQR)	6 (6, 7)	6 (6, 7)	6 (6, 7)
HIV tests provided (prior to study implementation)			
*Blood‐based HIVST*	31/40 (78)	21/24 (88)	10/16 (62)
*RDT*	19/40 (48)	15/24 (62)	4/16 (25)
*Oral‐fluid HIVST*	17/40 (42)	11/24 (46)	6/16 (38)
*No testing*	5/40 (12)	1/24 (4)	4/16 (25)
Counsel clients before and/or during HIV testing (*N* = 35)	32/35 (91)	21/23 (91)	11/12 (92)
Typical time spent counselling a client for an HIV test (*N* = 32)	20 (15, 43)	20 (15, 30)	30 (20, 45)

^a^ Behaviours self‐reported in the past 6 months.

^b^ Behaviours self‐reported in the past 3 days (i.e., 72 hours).

Among pharmacy providers, the median age was 31 years (IQR 27–37 years) and most 60% (24/40) were men (see Table 1). Almost half of the providers owned the pharmacy in which they worked (42%, 17/40) and had worked as a provider for a median of 6 years (IQR 4–10 years). Prior to study implementation, most providers delivered some form of HIV testing (88%, 35/40); the most common form was blood‐based HIVST (78%, 31/40), followed by RDT (without certification; 48%, 19/40), then oral‐fluid HIVST (42%, 17/40). Among those delivering HTS, almost all reported counselling clients before and during testing (91%, 32/35). Compared to female providers, more male providers delivered RDT (*p* = 0.02).

All 1500 enrolled clients completed pharmacy provider‐delivered HIVST and HTS counsellor‐administered RDT (see Figure 1). Among the HIV self‐tests interpreted by pharmacy providers, 94.5% (1417/1500) were negative and 5.5% (83/1500) were positive. Among the RDTs interpreted by HTS counsellors, 95.7% (1436/1500) were negative and 4.3% (64/1500) were positive. When the provider‐interpreted HIVST and HTS counsellor‐interpreted RDT results were discrepant (1.5%, 23/1500), the First Response and PCR test results were largely consistent with the RDT results (First Response: 87%, 20/23; PCR: 95%, 20/21).

**Figure 2:**
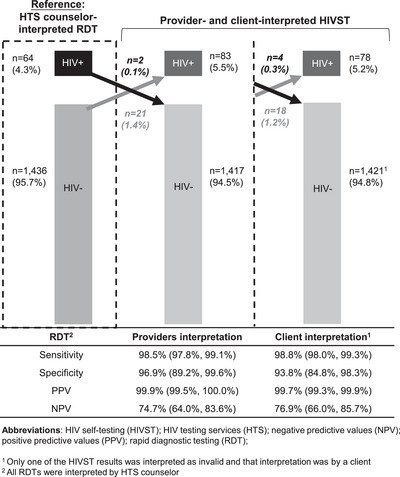
Performance of blood‐based HIV self‐testing compared to rapid diagnostic testing.

The overall performance of pharmacy provider‐delivered blood‐based HIVST was high (see Figure 2). The sensitivity of pharmacy provider‐interpreted HIVST was 98.5% (95% CI 97.8%, 99.1%) and the specificity was 96.9% (95% CI 89.2%, 99.6%), while the sensitivity of client‐interpreted HIVST was 98.8% (95% CI 98.0%, 99.3%) and the specificity was 93.8% (95% CI 84.8%, 98.3%). The PPVs of pharmacy provider‐delivered HIVST were also high among providers (99.9%, 95% CI 99.5%, 100%) and clients (99.7%, 95% CI 99.3%, 99.9%), while the NPVs were a bit lower among providers (74.7%, 95% CI 64.0%, 83.6%) and clients (76.9%, 95% CI 66.0%, 85.7%).

**Figure 3:**
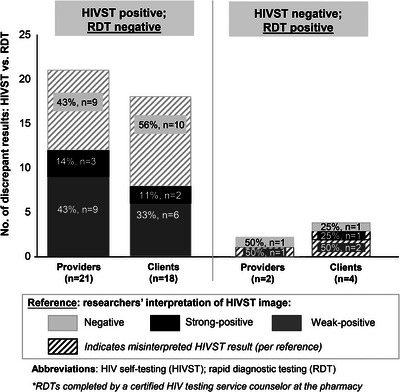
Poor performance of HIVST attributable to misinterpretation.

Misinterpretation of HIVST results by pharmacy providers and clients was a common reason for discrepant HIVST and RDT results (see Figure 3). Among the 23 discrepant provider‐interpreted HIVST and HTS counsellor‐interpreted RDT results, 43% (10/23) were likely attributable to provider HIVST misinterpretation; 43% (9/21) when the RDT was negative and 50% (1/2) when the RDT was positive. Then, among the 22 discrepant client‐interpreted HIVST and HTS counsellor‐interpreted RDT results, 59% (13/22) were likely attributable to client HIVST misinterpretation; 56% (10/18) when the RDT was negative and 75% (3/4) when the RDT was positive. This suggests the remaining discrepant HIVST and RDT test results were attributable to differences in test kit performance, with weak‐positive HIVST results comprising most of these discrepant results among providers (70%, 9/13) and clients (67%, 6/9).

During the HIVST process, most clients asked for assistance from pharmacy providers (79%, 1190/1500) (see Table 2). The most common steps where clients asked for assistance were using the lancet to puncture the skin (50%, 756/1500), collecting blood from the fingertip (56%, 838/1500) and adding the sample to the pad (41%, 615/1500). Among the clients who tested HIV positive (via RDT), 89% (57/64) planned to start treatment. Among the clients who tested HIV negative (via RDT), 39% (558/1436) planned to start PrEP; most of whom planned to start PrEP within the next week (43%, 242/558) or next month (43%, 242/558). Additionally, most clients planning to start PrEP preferred PrEP access at a public HIV clinic (62%, 346/558), followed by a private pharmacy (45%, 250/558). These experiences remained consistent across sex groups.

**Table 2:** Clients’ experiences with and perceptions of pharmacy‐delivered HIVST, *n*/*N* (%)

Pharmacy clients	All, *N* = 1500	Men, *N* = 546	Women, *N* = 954
**HIVST experience** ^a^			
Requested provider HIVST assistance	1190/1500 (79)	406/546 (74)	784/954 (82)
Steps clients needed assistance with:			
*Using lancet to puncture skin*	756/1190 (64)	233/406 (57)	523/784 (67)
*Collecting blood from fingertip*	838/1190 (70)	273/406 (67)	565/784 (72)
*Adding the sample to the pad*	615/1190 (52)	186/406 (46)	429/784 (55)
*Adding the chase buffer*	401/1190 (34)	135/406 (33)	266/784 (34)
*General handling of the test kit*	353/1190 (30)	127/406 (31)	226/784 (29)
*Preparing finger for blood collection*	316/1190 (27)	98/406 (24)	218/784 (28)
*Reading the instructions*	285/1190 (24)	80/406 (20)	205/784 (26)
Minutes provider spent with HIVST assistance, median IQR	2 (1–3)	2 (1–3)	2 (1–4)
**Linkage to care plans**			
Among those who tested HIV negative^b^			
Plans to start PrEP	558/1436 (39)	215/529 (41)	343/907 (38)
Among those who tested HIV positive^c^			
Plans to start ART	57/61 (93)	16/16 (100)	41/45 (91)
**Acceptability** ^d^ **of HIVST at the pharmacy** ***(TFA: component construct)***			
I am confident that I conducted the HIVST correctly *(TFA: self‐efficacy)*	1473/1500 (98)	543/546 (100)	930/954 (97)
I felt comfortable asking for provider assistance if needed *(TFA: self‐efficacy)*	1482/1500 (99)	536/546 (99)	946/954 (99)
I am confident that I interpreted HIVST results correctly *(TFA: self‐efficacy)*	1491/1500 (99)	543/546 (100)	948/954 (99)
It was hard to prick finger and collect blood for HIVST^e^ *(TFA: burden)*	835/1500 (56)	266/546 (49)	569/954 (60)
It was hard for me to read the results of the test^e^ *(TFA: burden)*	100/1500 (7)	39/546 (7)	61/954 (6)
Doing the HIV test interfered my other priorities^e^ *(TFA: opportunity costs)*	180/1500 (12)	80/546 (15)	100/954 (10)
HIVST did not create any moral/ethical problems *(TFA: ethicality)*	1463/1500 (98)	531/546 (97)	932/954 (98)
I believe the HIVST result was correct *(TFA: perceived effectiveness)*	1486/1500 (99)	540/546 (99)	946/954 (99)

**Abbreviations**: HIVST (HIV self‐testing); TFA (Theoretical Framework of Acceptability)

^a^ As reported by research assistants observing pharmacy provider‐delivered HIVST at the study pharmacies.

^b^ In this study, 1436 clients tested HIV‐negative, including 529 male clients and 907 female clients.

^c^ In this study, 61 clients tested HIV positive, including 16 male clients and 45 female clients.

^d^ Percentage of participants that agreed or strongly agreed to each statement, measured using a 5‐point Likert scale.

^e^ Statements reverse coded.

**Figure A1:**
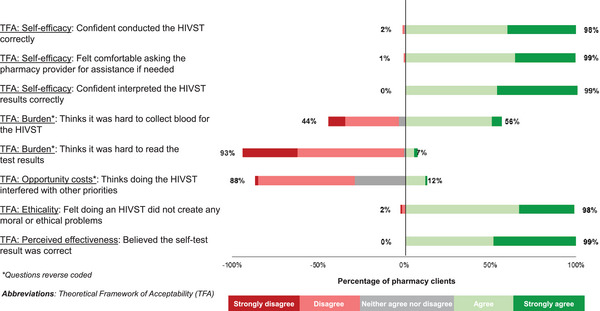
The acceptability of HIVST among clients (*N* = 1500).

Clients perceived pharmacy‐based HIVST as highly acceptable (see Table 2 and Figure A1 for details). Almost all clients (>97%) agreed or strongly agreed to most statements assessing the different acceptability component constructs [39] of the intervention. For example, 98% (1473/1500) of clients reported they felt confident they conducted the HIVST correctly (TFA: affective attitude) and 99% (1486/1500) believed the HIVST results (TFA: perceived effectiveness). Additionally, only 7% (100/1500) of clients found it hard to read the HIVST result (TFA: burden) and only 12% (180/1500) thought HIVST at the pharmacy interfered with their other priorities (TFA: opportunity costs). The only component construct that did not meet our acceptability threshold was a statement about the burden associated with collecting a blood sample for HIVST; 56% (835/1500) of clients reported that finger pricking for HIVST was hard, consistent with our observations of clients using HIVST. These acceptability findings remained consistent across sex groups.

## 4 DISCUSSION

The performance of provider‐delivered blood‐based HIVST at private pharmacies was high compared to RDT, the standard of care for HIV PrEP dispensing in Kenya. In this study, the sensitivity of the HIVST, compared to RDT, was >98% when interpreted by both providers and clients and the specificity was >96% when interpreted by providers and >93% when interpreted by clients. In practice, pharmacy providers delivering PrEP would ultimately need to interpret the HIVST results to confirm HIV‐negative status prior to PrEP dispensing. Among the discrepant HIVST and RDT test results, misinterpretation of HIVST results was attributable to roughly half of the discrepant tests; enhanced provider HIVST training and support tools (i.e. instructional videos and improved job aids) could potentially improve provider‐delivered HIVST performance.

When considering the use of HIVST to support PrEP initiation at pharmacies and other community‐based settings, HIVST specificity is the most important consideration. False HIV‐negative test results and inappropriate PrEP initiation among persons living with HIV could potentially lead to the development of drug resistance, which could be transmitted and decrease the effectiveness of PrEP and first‐line ART regimens [41, 42]. Several modelling studies, however, have projected that the potential reductions in HIV incidence associated with expanded PrEP coverage using HIVST likely outweigh the risk of drug resistance [6, 43]. We also found high HIVST specificity with pharmacy provider‐delivered HIVST. However, we did observe provider misinterpretation of faint HIV‐positive lines, which can be associated with acute HIV acquisition. The potential for PrEP‐associated drug resistance development is particularly high during acute HIV acquisition [44], so monitoring HIVST use for PrEP delivery and enhanced training for providers is likely needed for accurate HIV diagnosis and referral to ART.

Our findings suggest that using provider‐delivered HIVST for PrEP delivery is likely to have a similar performance as RDT. HIVST for PrEP delivery in community‐based settings is a promising strategy to expand PrEP coverage and facilitate continuation. The SEARCH trial demonstrated that offering participants choices for HIV prevention, including HIVST, substantially increased coverage of biomedical prevention interventions during periods of HIV risk [45]. Additionally, HIVST was preferred over RDT in the SEARCH trial, with the majority (71%) of participants in the intervention arm selecting this testing option [46]. A discrete choice experiment evaluating user preferences for PrEP service delivery through online pharmacies in Kenya also found that participants strongly preferred HIVST over RDT [47]. The greater privacy offered by HIVST may overcome barriers associated with RDT, including confidentiality and stigma concerns [48], and increase PrEP coverage among populations that could benefit.

Our findings additionally suggest that populations with behaviours associated with HIV risk frequently attend private pharmacies and could be reached by pharmacy‐delivered PrEP services. Even in high HIV prevalence settings, like western Kenya [31], most individuals who test for HIV test negative. As knowledge of HIV status increases, the prevalence of undiagnosed HIV is relatively low. When individuals at risk of HIV are referred to the clinic for PrEP services, few are likely to follow through on this referral [49]. In this study, for example, less than half of pharmacy clients who tested HIV negative planned to visit a clinic for PrEP, while most clients who tested HIV positive planned to initiate ART. Among the clients who planned to start PrEP, public clinics were largely reported as the preferred setting for PrEP access likely due to selection bias in those asked this question (i.e. those not interested in PrEP initiation at public clinics were not asked this question). If PrEP services were to be expanded to private pharmacies, evidence from the pilot studies in Kenya suggests that individuals not otherwise interested in PrEP access at public clinics might engage in PrEP services [21, 22, 49, 50, 51, 52].

This study has several limitations. First, all pharmacy providers and clients were observed by a trained research assistant during the HIVST process, which may have increased their performance and positively biased our outcomes. Second, all pharmacy providers completed a 1‐day training on HIVST use and interpretation, and clients and providers received job aids (e.g. flipbooks) to facilitate the HIVST process, which could have increased their performance and limit the generalizability of our findings. However, these are relatively simple and low‐cost interventions that could be easily scaled (i.e. HIV RDT job aids are commonly found at public clinics) and are feasible for pharmacy providers to complete among their other competing priorities. A 1‐day training, for example, is substantially shorter than the 2‐week training required for RDT provision in Kenya. Third, we did not capture what sexual and reproductive health products clients were seeking at their pharmacy visit. Finally, we only assessed the acceptability of pharmacy‐delivered HIVST services among clients who agreed to enroll in our study and complete HIV testing at the participating pharmacies, which could have positively biased our acceptability findings. We did not measure the acceptabilty of pharmacy‐delivered HIVST services among pharmacy providers.

## 5 CONCLUSIONS

HIVST is a promising tool that has been underutilized to support health systems [53]. The use of HIVST over RDT in private pharmacies has advantages, including shorter provider training requisites, increased client empowerment, and increased health system efficiencies. Potential disadvantages, however, may include higher costs which should be explored in further economic analyses. Global policymakers should consider HIVST to support differentiated community‐based PrEP delivery models, like pharmacy‐based PrEP delivery, that can expand PrEP reach to individuals who could benefit and bring us closer to ending the AIDS epidemic.

## COMPETING INTERESTS

None of the authors have any competing interests to declare.

## AUTHORS’ CONTRIBUTIONS

KFO, DKW, EAB and KN contributed to the study conception and design of this HIVST performance study. BK, PAO and VO led recruitment and study operations with support from RCM and KH. MA, SZ and KFO analysed the data and KFO wrote the first draft of this manuscript. All authors edited the draft, provided insights and approved the final manuscript for publication.

## FUNDING

This work was supported by the Bill & Melinda Gates Foundation (BMGF, INV‐033052). KFO received additional funding from the National Institute of Mental Health (R00 MH121166).

## Data Availability

The data that support the findings of this study are available on request from the corresponding author. The data are not publicly available due to privacy or ethical restrictions.
